# Supplementary material for: Beyond Blacklists: A Critical Assessment of Exclusion Set Generation Strategies and Alternative Approaches
Source: bioRxiv. 2025 Feb 8:2025.02.06.636968. Preprint. [Version 1] doi: 10.1101/2025.02.06.636968 (PMC11839099; doi:10.1101/2025.02.06.636968)
Supplement: Supplementary file 1 [file media-1.pdf]

Beyond Blacklists: A Critical Assessment of Exclusion Set Generation Strategies and Alternative Approaches

Supplementary Figures

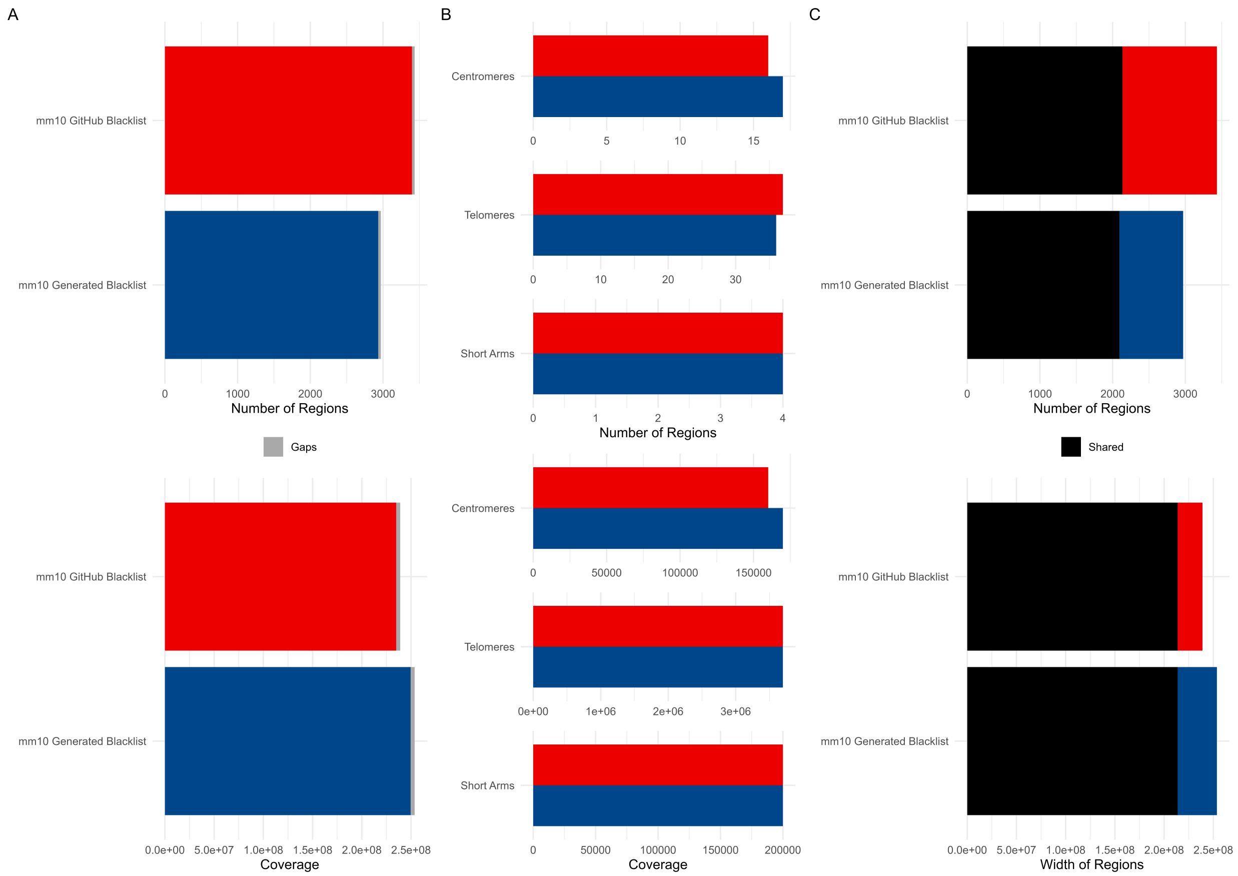

Supplementary Figure S1. Differences between the GitHub version of the mm10 exclusion set and our mm10 exclusion set generated with the Blacklist software. (A) Differences in count, coverage, and width distribution. (B) Differences in gap coverage. (C) Pairwise overlaps of region counts and genome coverage between sets.

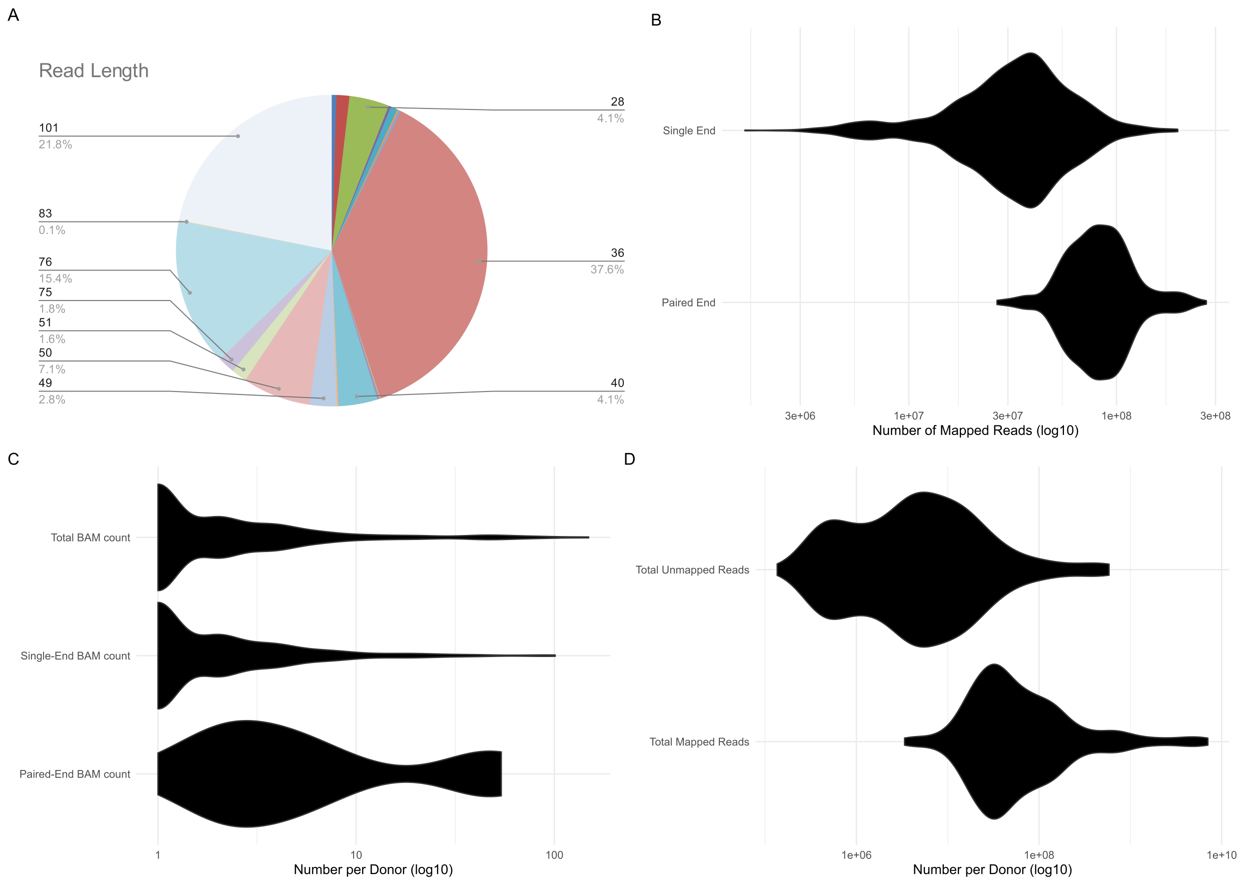

Supplementary Figure S2. The diversity of BAM files used to generate the hg38 exclusion set. (A) Read length proportions among 1,225 BAM files. (B) Number of mapped reads for single- and paired-end files. (C) Number of single- and paired-end files per donor. (D) Total number of mapped and unmapped reads per donor.

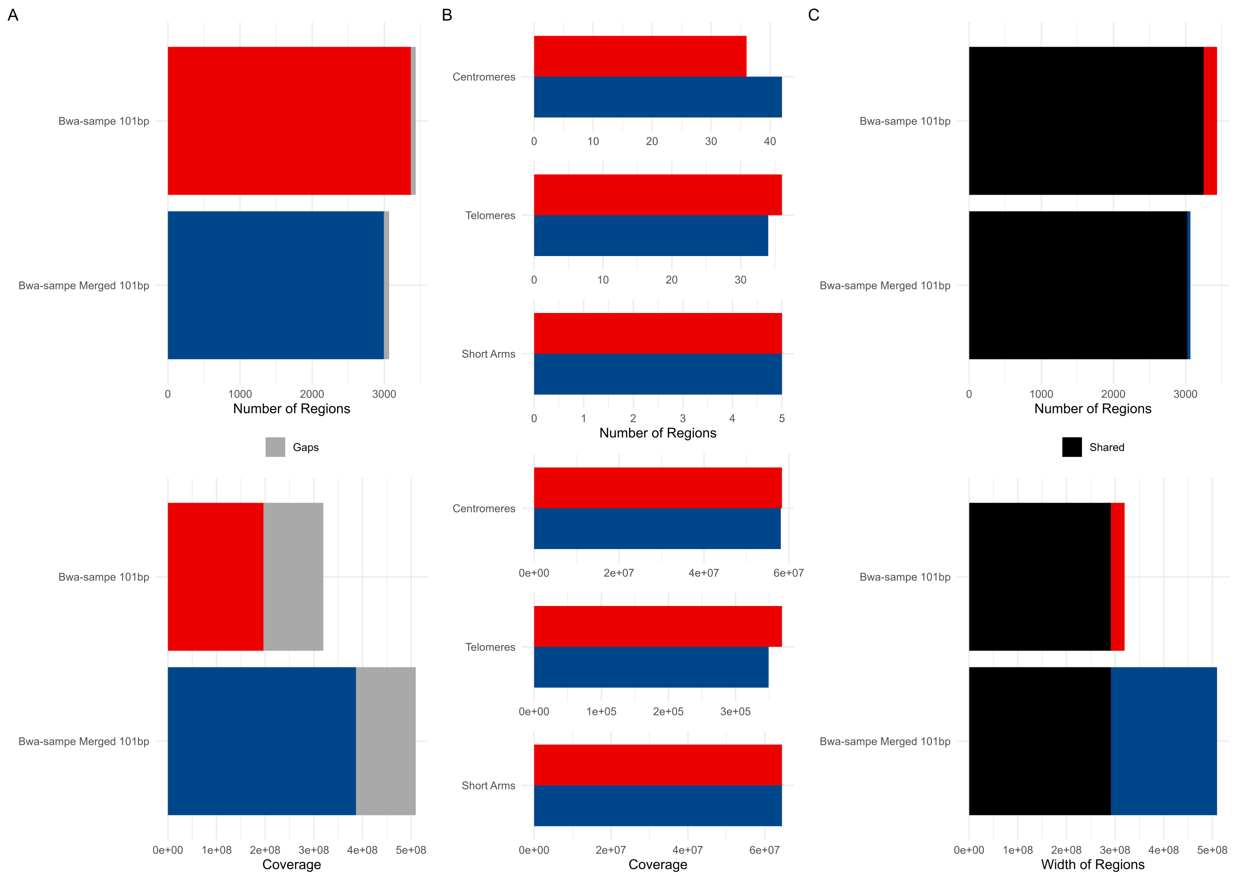

Supplementary Figure S3. Exclusion set characteristics generated from the 274 unmerged 101bp BAM files and the corresponding merged BAM file. (A) Differences in count, coverage, and width distribution. (B) Differences in gap coverage. (C) Pairwise overlaps of region counts and genome coverage between sets.

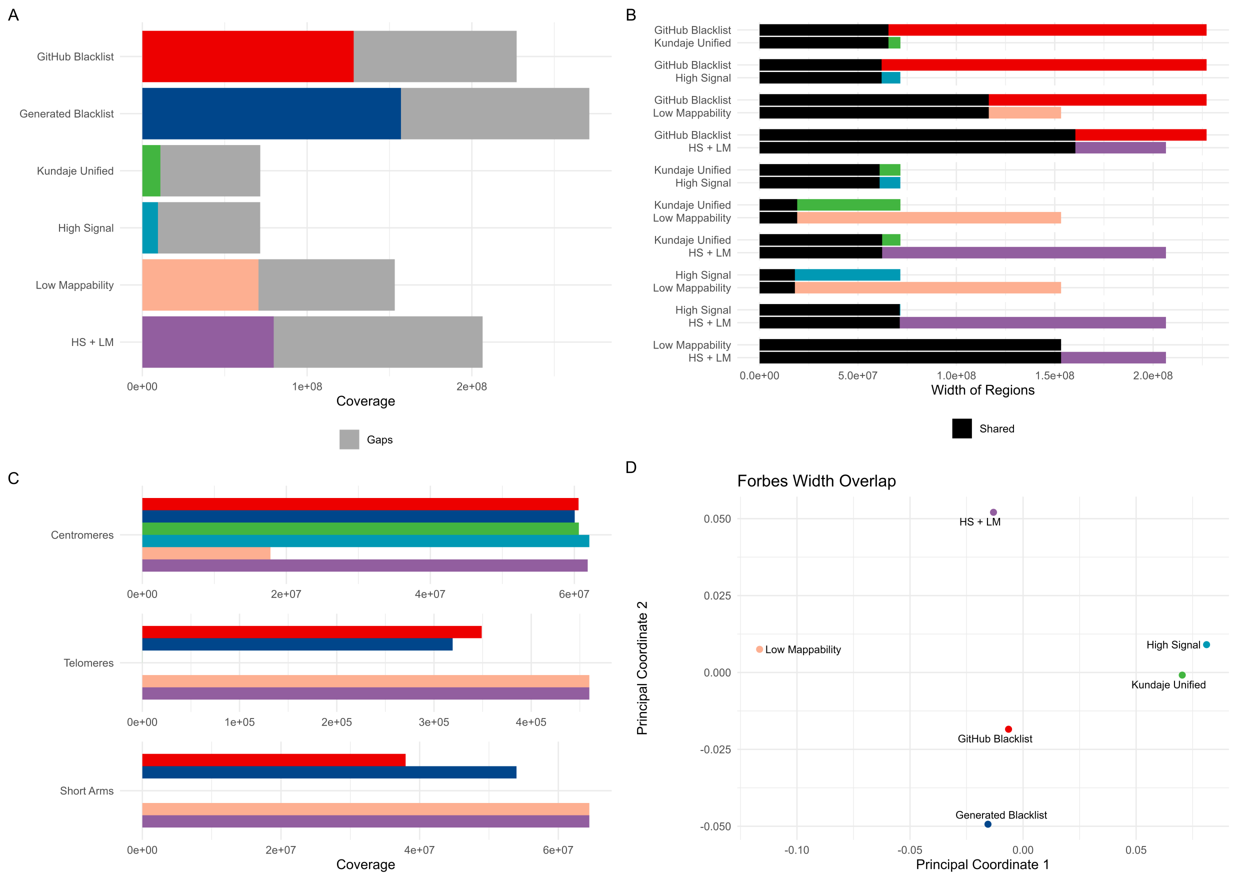

Supplementary Figure S4. Differences between the GitHub version of the hg38 exclusion set and manually defined gold-standard exclusion sets. (A) Differences in width. (B) Pairwise width overlaps between the GitHub exclusion set and manually defined gold-standard exclusion sets. (C) Differences in gap coverage. (D) Multidimensional scaling plot of Forbes width similarity among the GitHub exclusion set and manually defined gold standards.

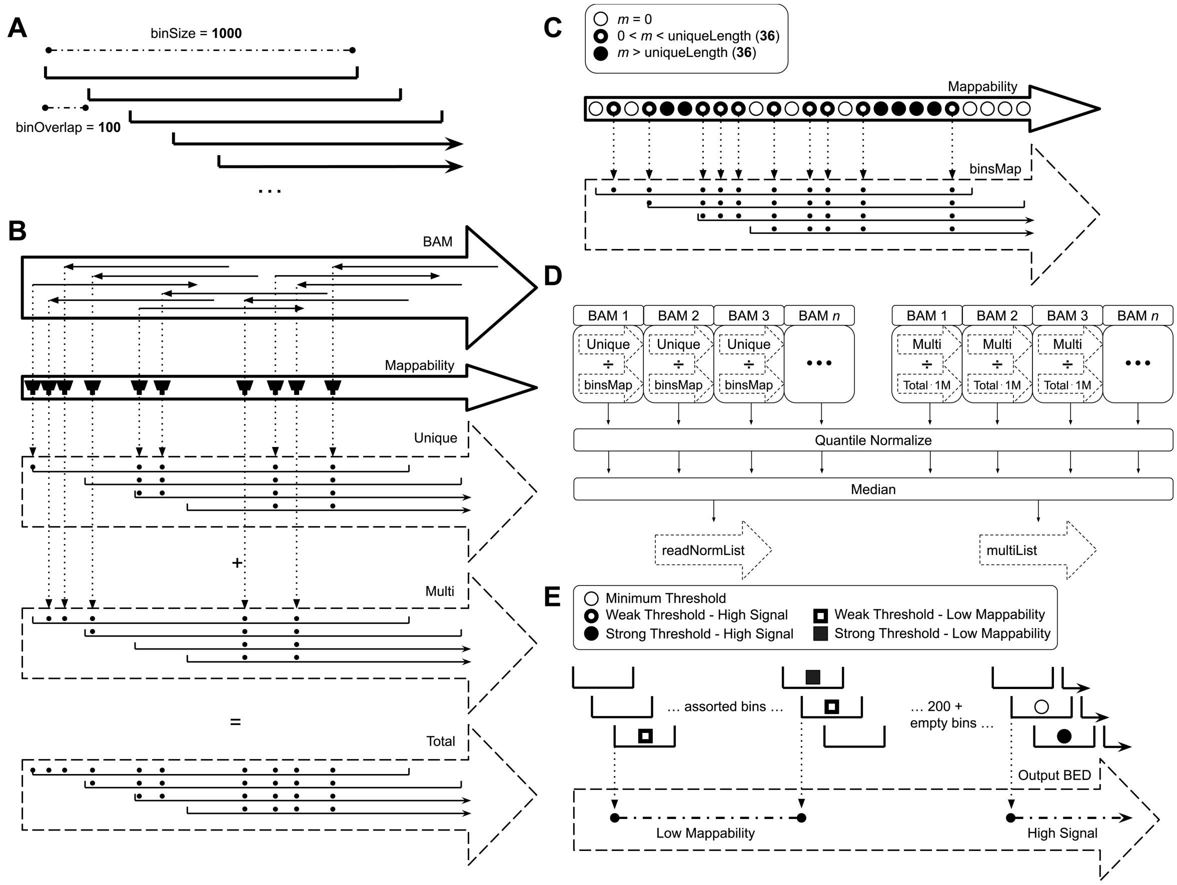

Supplementary Figure S5. The Blacklist Algorithm. A) **Binning parameters:** Two internal parameters, "binSize" and "binOverlap," define how bins are created. B) **Read sorting:** The start position of each read is sorted according to the read's length and compared using the given pre-calculated mappability vector. C) **The "binsMap" vector:** This vector is generated using the "uniqueLength" threshold. D) **Normalization:** The signal ("Unique") and mappability ("Multi") vectors are divided, quantile normalized, and the median is taken per bin. E) **Region calling:** This step uses several predefined internal parameters/thresholds to call and annotate excludable regions.

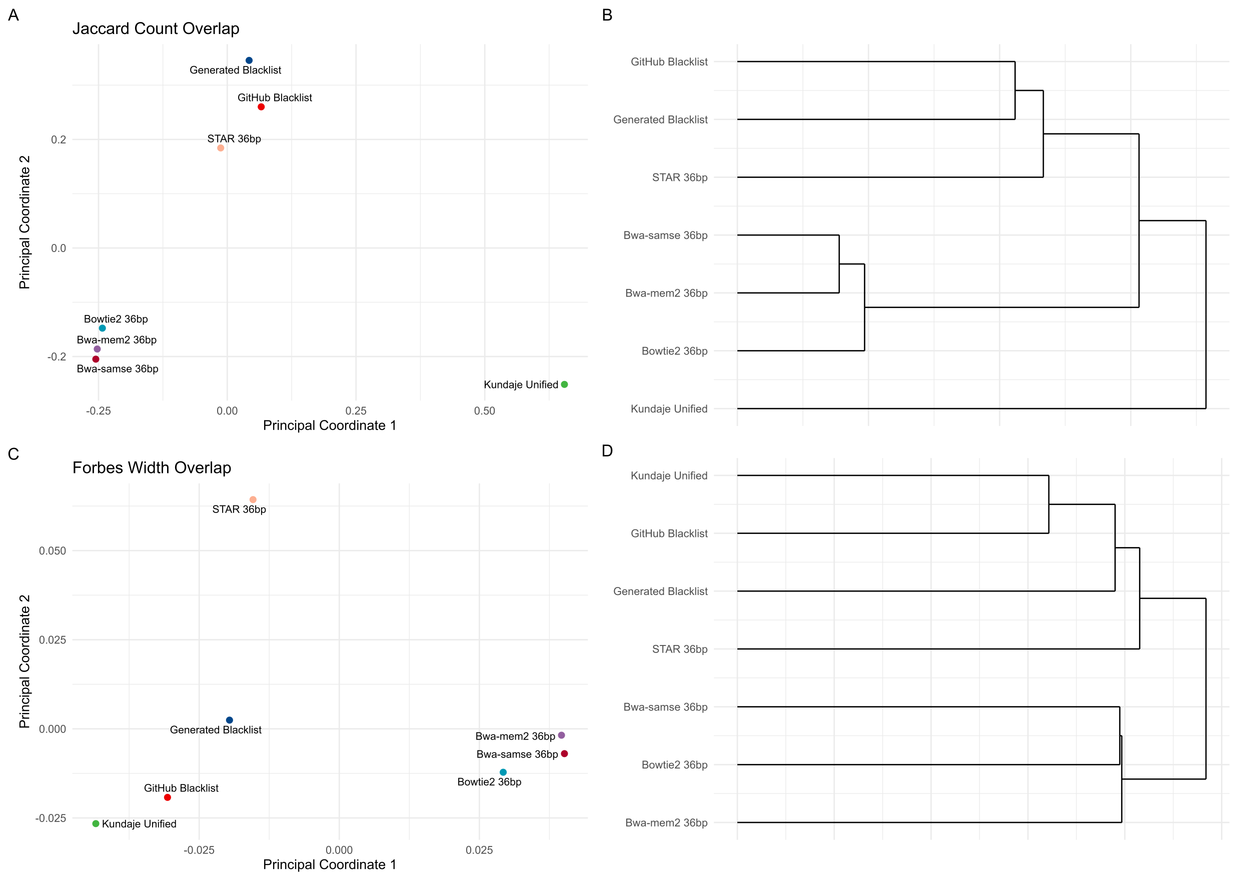

Supplementary Figure S6. Relationships among realigned 36bp exclusion sets. A, B) Jaccard count overlap, multidimensional scaling, and hierarchical clustering; C, D) Forbes width overlap, MDS, and clustering.

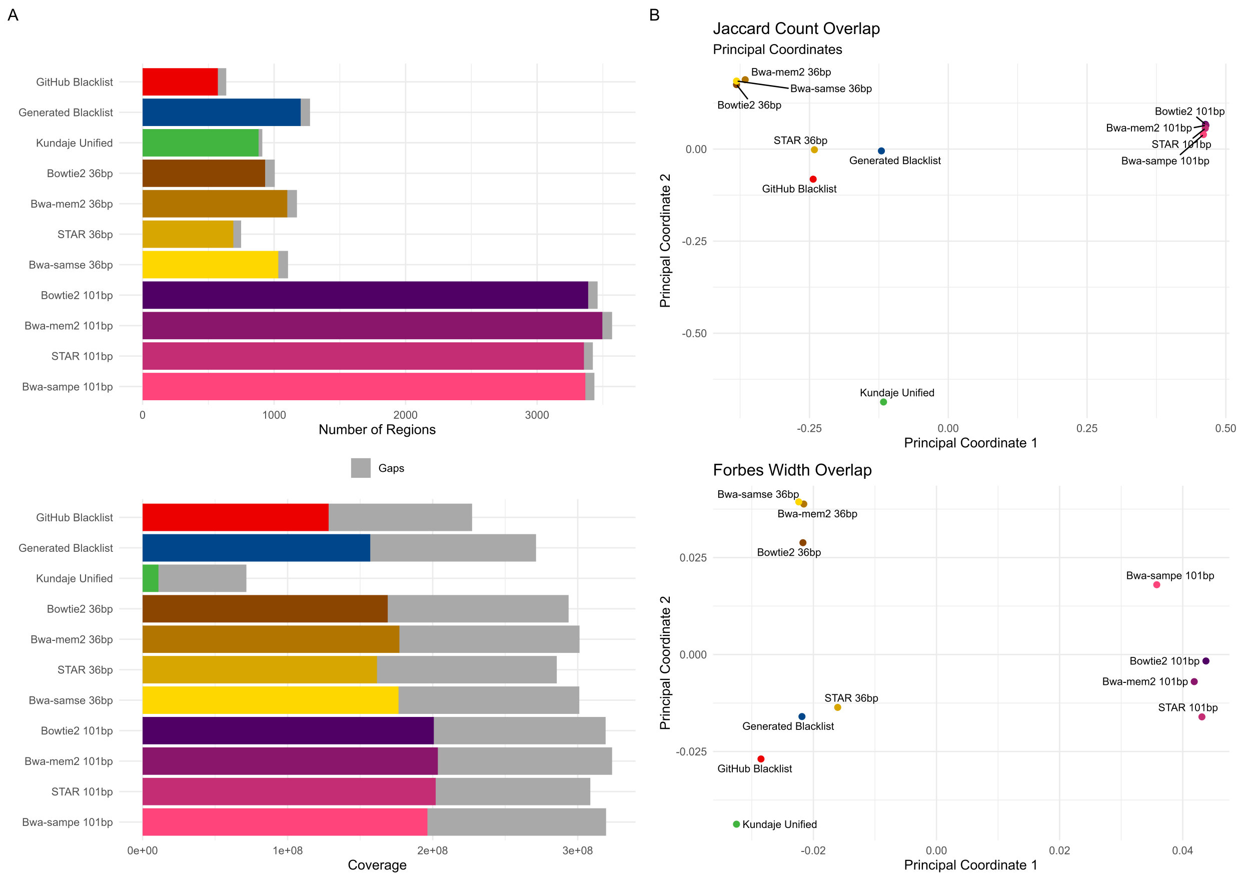

Supplementary Figure S7. Relationships among realigned 36bp and 101bp exclusion sets. A) Number and width distribution; B) Jaccard count overlap and Forbes width overlap MDS plots.

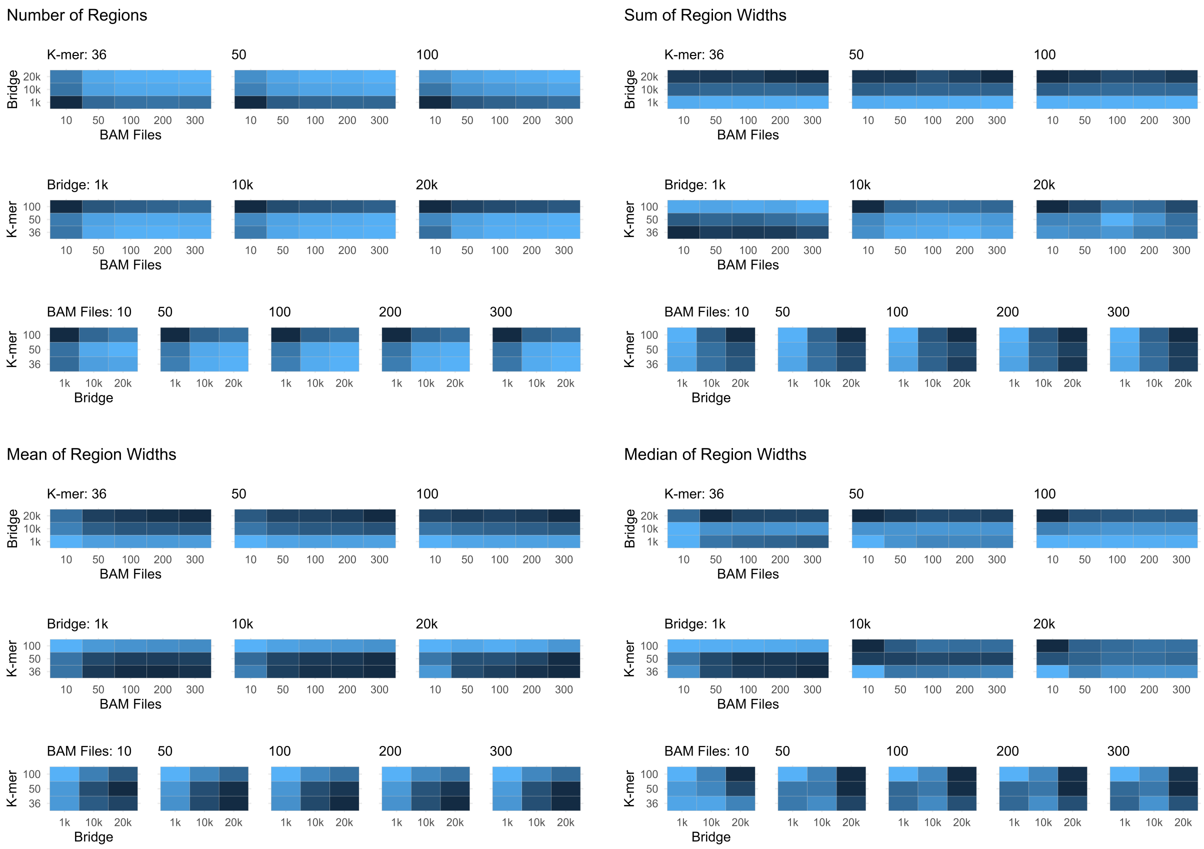

Supplementary Figure S8. The effect of varying the number of BAM files, the "bridge" parameter, and the "k-mer" parameter on the output of the Blacklist software. The effect on the number of exclusion regions, sum of region widths, and mean and median region widths.

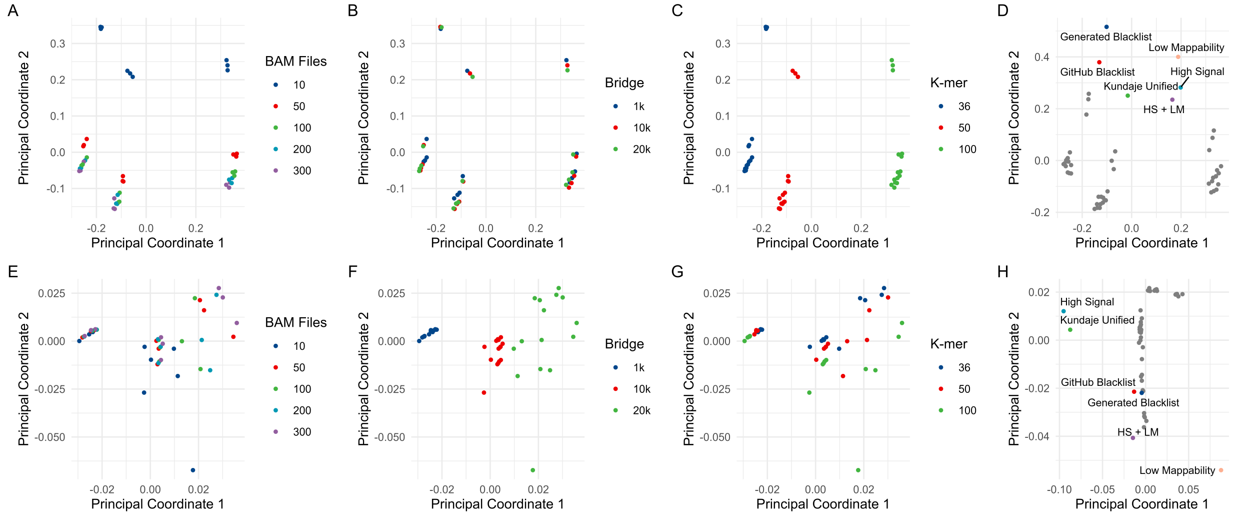

Supplementary Figure S9. Jaccard count overlap (A-D) and Forbes similarity (E-H) MDS plots colored by parameters. A), E) Number of BAM files; B), F) Bridge parameter; C), G) k-mer parameter; D), H) Parameter sweep results (gray) vs. reference exclusion sets.

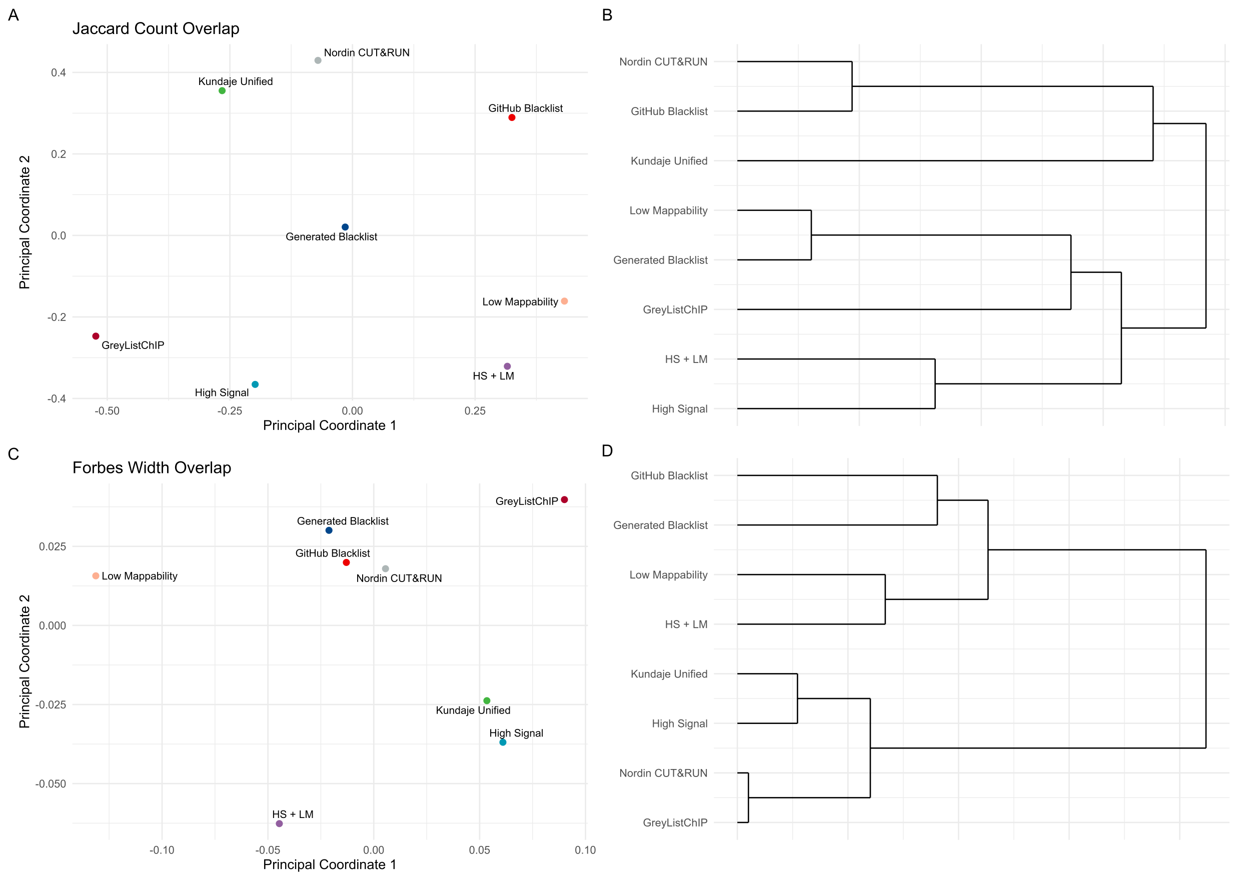

Supplementary Figure S10. Relationships among Blacklist, manually created, and Nordin CUT&RUN and GreyListChIP exclusion sets. A, B) Jaccard count overlap, multi-dimensional scaling, and hierarchical clustering; C, D) Forbes width overlap, MDS, and clustering.

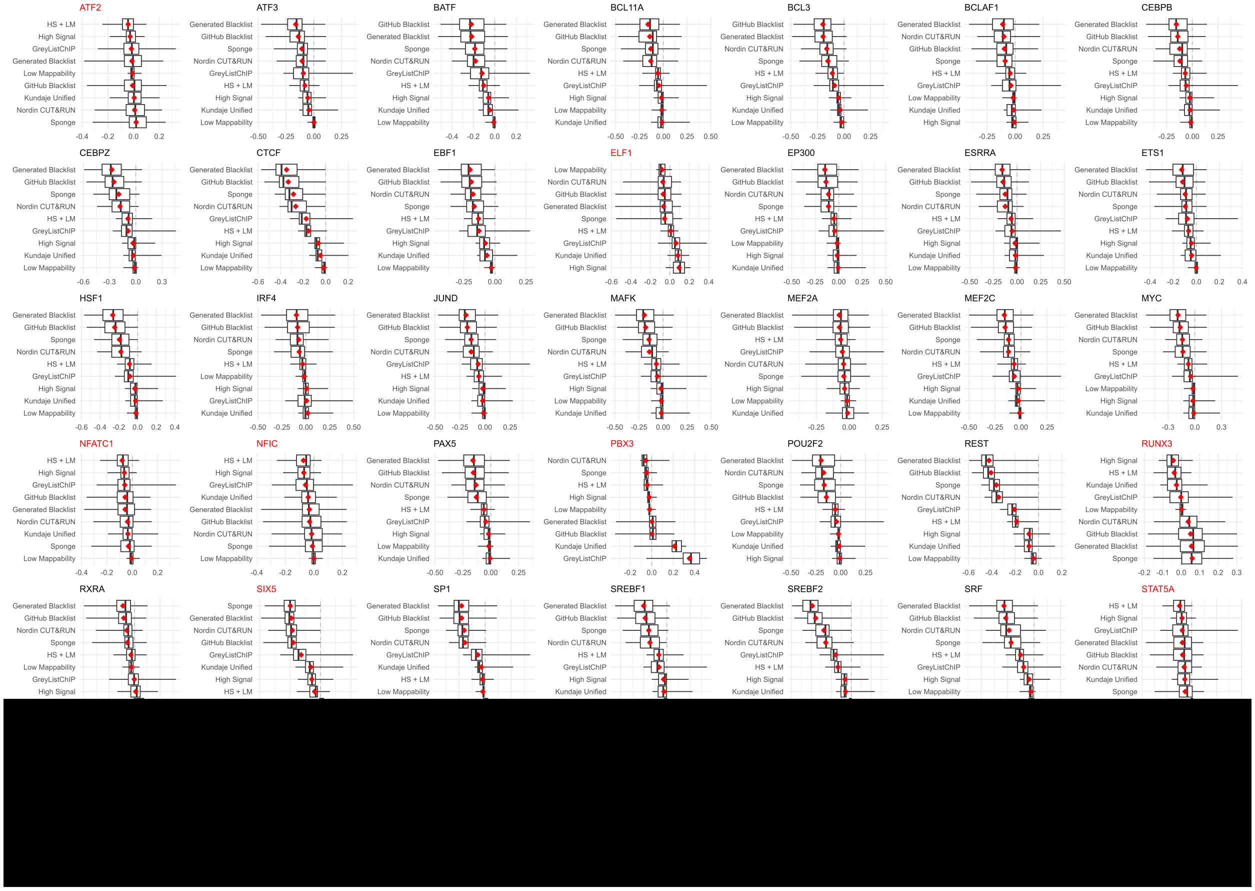

Supplementary Figure S11. Transcription factor-specific changes in ChIP-seq signal correlation with and without reads overlapping exclusion sets or aligned to the sponge. Data for the Gm12878 cell line is shown. Correlation difference distributions, red transcription factor names highlights cases where exclusion lists other than "Blacklist" and "Sponge" have the strongest decrease in correlations.

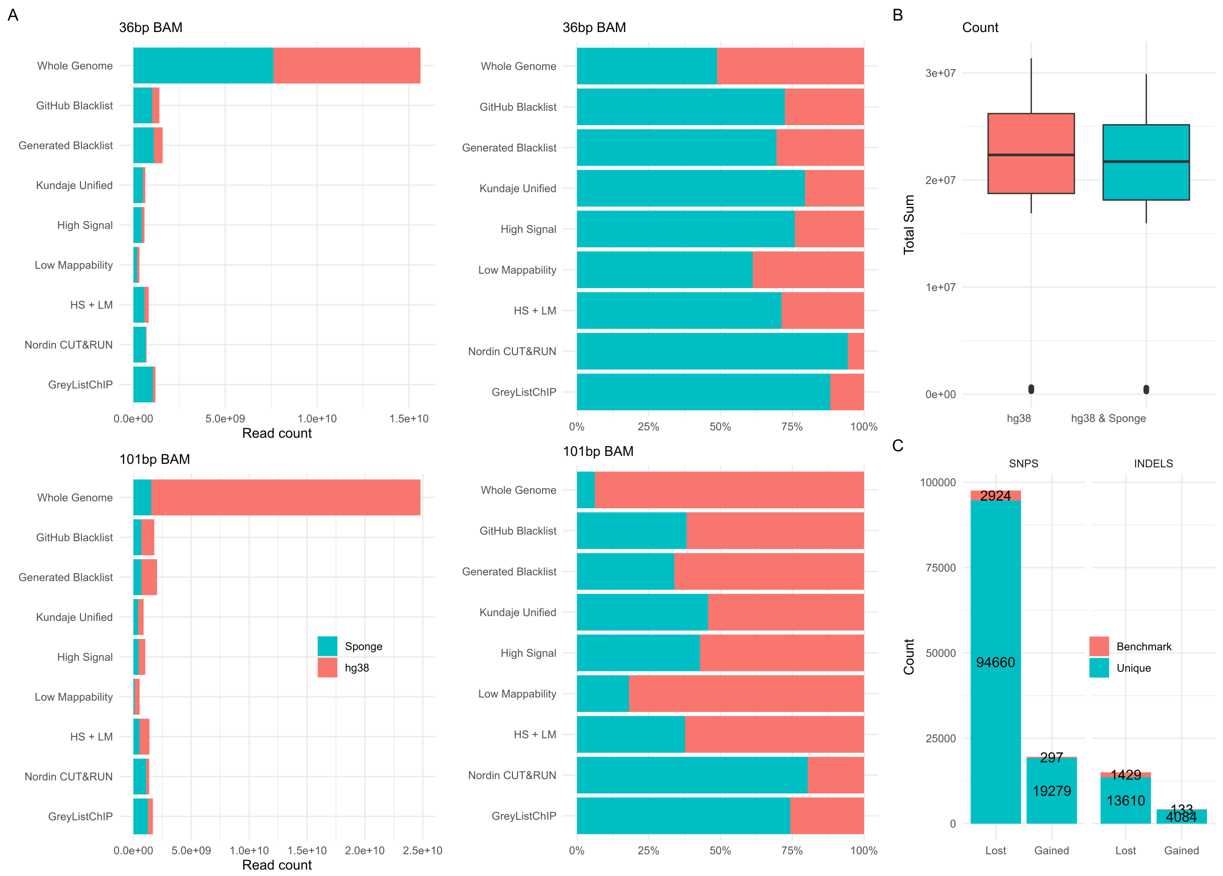

Supplementary Figure S12. The effect of "sponge" sequences on alignment. A) The number and proportion of 36 bp and 101 bp reads aligned to "sponge" sequences from the whole genome and from various excludable sets; B) The total number of reads overlapping annotated gene regions when aligned with and without the "sponge" sequences; C) The number of lost and gained SNPs and indels after aligning Gm12878 whole genome sequencing data to
