# Supplementary material for: Beyond Blacklists: A Critical Assessment of Exclusion Set Generation Strategies and Alternative Approaches
Source: bioRxiv. 2025 Feb 8:2025.02.06.636968. Preprint. [Version 1] doi: 10.1101/2025.02.06.636968 (PMC11839099; doi:10.1101/2025.02.06.636968)
Supplement: Supplementary file 2 [file media-2.pdf]

Beyond Blacklists: A Critical Assessment of Exclusion Set Generation Strategies and Alternative Approaches

Supplementary Note

Blacklist algorithm description

The Blacklist software takes as input one or more BAM files and a pre-calculated binary uint8 file representing mappability across a reference genome. The script then uses two internally defined parameters, "binSize" (the size of the genomic intervals; 1 kbp by default) and "binOverlap" (the amount of overlap between adjacent bins; 100 bp by default), to divide the genome into overlapping bins of a fixed size (Supplementary Figure S5A). These binning parameters ultimately determine the resolution of the resulting output regions.

The program then iterates through each genomic position in each BAM file and counts the start positions of reads for each bin. Three new vectors are created by comparing the read length to the uint8 mappability vector. The first vector represents mappable reads per bin, the second represents multi-mappable reads per bin, and the third is the total reads per bin (Supplementary Figure S5B). Another vector, "binsMap," is generated by counting the mappability of each position per bin if the read length falls between 0 and another internal variable, "uniqueLength" (intended to represent a homogeneous k-mer size; 36 by default), non-inclusively (Supplementary Figure S5C).

Next, the algorithm uses the binned vectors generated for each BAM file and creates two summary vectors: one estimating signal and one estimating mappability. To create the signal vector, the algorithm divides the individual vector representing binned counts of mappable reads by the global "binsMap" vector. Then, it applies a version of quantile normalization and takes the median across bins of equivalent positions. This process results in a single binned vector estimating signal. The mappability estimation follows a similar approach: for each BAM file, the algorithm divides the binned vector representing counts of multi-mappable reads by the binned vector representing total reads, then scales by one million. Like the signal vector, this data is quantile normalized, and the median value is taken, yielding a single binned vector estimating mappability.

The algorithm defines five internal thresholds that classify regions: weak and strong thresholds for both high signal and low mappability, and a fifth threshold that represents the minimum value of all bins in the signal vector. The weak and strong thresholds are set using percentile cutoffs: the 99th percentile for weak and the 99.9th percentile for strong. Once the cutoff values are established by taking these percentiles of both the binned signal and mappability vectors, the algorithm uses these cutoffs to call the resulting output regions.

This process starts at the first bin, comparing the cutoffs for both the signal and mappability vectors, and proceeds sequentially through each bin. If a bin meets or exceeds the weak threshold and is equal to the minimum threshold (specifically for the signal vector), the algorithm records the current bin's position as the start of a potential output region. If a later bin surpasses any strong threshold, the algorithm flags the marked region as ready to be output and continues to subsequent bins to extend the region. Once a predetermined number of consecutive bins (200 bins by default, equivalent to 20 kbp) fail to meet any threshold, or the last bin is processed, the algorithm either outputs the region ending at the last bin that passed any threshold or skips the region if a strong threshold was not met.

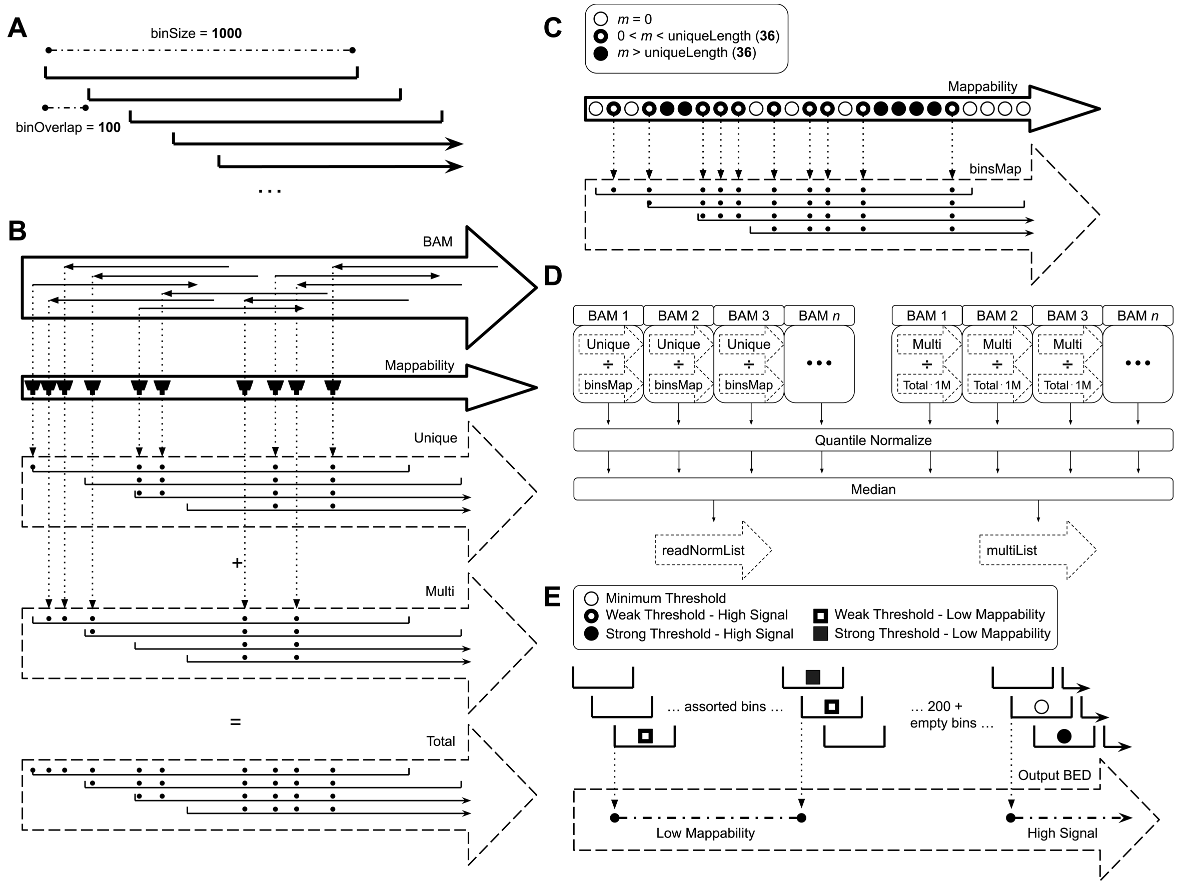

Supplementary Figure S5. The Blacklist Algorithm. A) **Binning parameters:** Two internal parameters, "binSize" and "binOverlap," define how bins are created. B) **Read sorting:** The start position of each read is sorted according to the read's length and compared using the given pre-calculated mappability vector. C) **The "binsMap" vector:** This vector is generated using the "uniqueLength" threshold. D) **Normalization:** The signal ("Unique") and mappability ("Multi") vectors are divided, quantile normalized, and the median is taken per bin. E) **Region calling:** This step uses several predefined internal parameters/thresholds to call and annotate excludable regions.
